# Influence of Clinical and Tumor Factors on Interfraction Setup Errors With Rotation Correction for Vacuum Cushion in Lung Stereotactic Body Radiation Therapy

**DOI:** 10.3389/fonc.2021.734709

**Published:** 2021-10-21

**Authors:** Hua Chen, Lingxiang Liu, Hao Wang, Yan Shao, Hengle Gu, Yanhua Duan, Aihui Feng, Ying Huang, Zhiyong Xu

**Affiliations:** ^1^ Radiation Oncology, Shanghai Chest Hospital, Shanghai Jiaotong University, Shanghai, China; ^2^ Department of Oncology, Guangzhou Panyu Central Hospital, Guangzhou, China

**Keywords:** vacuum cushion, SBRT, NSCLC, interfraction, setup error, clinical and tumor factors

## Abstract

**Purpose:**

To explore the influence of clinical and tumor factors over interfraction setup errors with rotation correction for non-small cell lung cancer (NSCLC) stereotactic body radiation therapy (SBRT) patients immobilized in vacuum cushion (VC) to better understand whether patient re-setup could further be optimized with these parameters.

**Materials and Methods:**

This retrospective study was conducted on 142 NSCLC patients treated with SBRT between November 2017 to July 2019 in the local institute. Translation and rotation setup errors were analyzed in 732 cone-beam computed tomography (CBCT) scans before treatment. Differences between groups were analyzed using independent sample t-test. Logistic regression test was used to analyze possible correlations between patient re-setup and clinical and tumor factors.

**Results:**

Mean setup errors were the largest in anterior–posterior (AP) direction (3.2 ± 2.4 mm) compared with superior–inferior (SI) (2.8 ± 2.1 mm) and left–right (LR) (2.5 ± 2.0 mm) directions. The mean values were similar in pitch, roll, and rtn directions. Of the fractions, 83.7%, 90.3%, and 86.6% satisfied setup error tolerance limits in AP, SI, and LR directions, whereas 95% had rotation setup errors of <2° in the pitch, roll, or rtn directions. Setup errors were significantly different in the LR direction when age, body mass index (BMI), and “right *vs.* left” location parameters were divided into groups. Both univariate and multivariable model analyses showed that age (p = 0.006) and BMI (p = 0.002) were associated with patient re-setup.

**Conclusions:**

Age and BMI, as clinical factors, significantly influenced patient re-setup in the current study, whereas all other clinical and tumor factors were not correlated with patient re-setup. The current study recommends that more attention be paid to setup for elderly patients and patients with larger BMI when immobilized using VC, especially in the left–right direction.

## Background

Local tumor control rates of stereotactic body radiotherapy (SBRT) in early-stage non-small cell lung cancer (NSCLC) is approximately 90%, with survival rates matching those of surgery in similar patient groups (1, 2). SBRT has become the standard non-surgical treatment of choice for patients with NSCLC who are not scheduled for operation (3–6). Due to high fractional dose within the target, steep dose fall-off outside the tumor, and few treatment fractions, a highly accurate and reproducible patient setup is critical during treatment, especially in NSCLC, where tumor movements are largely influenced by respiration movement (7). If the target location is slightly off, it may lead to insufficient target coverage or overdose to organs at risk (OAR).

Accurate patient positioning for lung SBRT is mainly undertaken using image-guided radiation therapy (IGRT) combined with an immobilization device. Studies have reported more accurate setup comprising cone-beam computed tomography (CBCT) scans compared with electronic portal imaging device (8). In addition, soft tissue match on either anatomical landmarks or primary tumor match for CBCT could have higher accuracy compared with bones match (9–11). Therefore, daily CBCT image guidance with soft-tissue setup is recommended in lung SBRT (12–14). Previous studies indicated that immobilization device is also an important strategy to ensure reproducible patient setup. Moreover, several guidelines for SBRT immobilization devices have been reported, and different immobilization systems generate different inter- and intrafraction errors (15–17). However, no clear standard approach has been reported so far (16, 18, 19), indicating that each institute needs to select an immobilization approach based on the best available evidence and characteristics of the institute (17).

Vacuum cushion (VC) is a commonly used immobilization device in SBRT, which is widely adopted for lung SBRT in our institution, where setup shifts are undertaken in combination with VC and 6D couch. A few previous studies compared VC with other devices, including thermoplastic masks (15) and abdominal compression (20). However, no studies indicating whether there were significant differences in setup error magnitude under the same fixation conditions like VC among patients with other different conditions have been reported. Furthermore, factors influencing the setup error with rotation correction of VC are not yet known.

Therefore, the purpose of the current study was to explore effects of clinical and tumor factors on interfraction setup errors with rotation correction for lung SBRT patients immobilized with VC. The current study hypothesized that translation and rotation setup errors are correlated with clinical and tumor factors.

## Material and Methods

### Patient Data

In the present study, medical records of NSCLC patients who underwent lung SBRT in Shanghai Chest Hospital from November 2017 to July 2019 were reviewed. Inclusion criteria included patients with early-stage I NSCLC (T1 or T2, N0, M0) or oligometastatic lung cancer and immobilized using VC. A total of 142 patients were selected for inclusion in the current study. This retrospective study was approved by the Ethics Committee of Shanghai Chest Hospital.

**Tables 1** and **2** summarize general information and detailed clinical and tumor characteristics of the included patients. **Table 1** shows clinical characteristics including age, gender, body mass index (BMI), ability to understand Mandarin, and education level. The average age was 68 ± 11 years, ranging from 37 to 88 years. A total of 110 patients could speak Mandarin, whereas 32 patients could not speak Mandarin and needed family members to accompany them when positioning.

**Table 1:** Clinical characteristics for patients (n = 142).

Patient info	n
**Age (years)**	
Mean	68 ± 11
Range	37–88
**Gender**	
Male	85
Female	57
**BMI**	
BMI ≤ 18.5	12
18.5 < BMI ≤ 24	75
24 < BMI	55
**Ability to understand Mandarin**	
Yes	110
No	32
**Education level**	
Illiterate	22
Primary school	33
Middle school	35
High school	33
University qualifications	19

BMI, body mass index.

**Table 2:** Tumor characteristics for patients (n = 142).

Tumor parameters	Value	Range
**Location**		
Right	74	–
Left	68	–
**Location**		–
Upper	75	–
Middle	20	–
Lower	47	–
**Maximum diameter of ITV (cm)**	2.6 ± 0.8	1–5.3
**Volume of ITV (cm^3^)**	11.65 ± 12.41	0.54–75.88
**Distance from ITV boundary to vertebral body boundary (cm)**	3.9 ± 2.6	0–11.6
**Distance from ITV boundary to heart boundary (cm)**	3.8 ± 2.0	0–8.7
**Distance from ITV boundary to chest wall boundary (cm)**	0.8 ± 1.2	0–5.7

ITV, internal target volume.

Tumor characteristics including tumor location, maximum diameter of internal target volume (ITV), volume of ITV, distance from ITV boundary to vertebral body boundary, distance from ITV boundary to heart boundary, and distance from ITV boundary to chest wall boundary are listed in **Table 2**.

### Simulation, Contouring, and Treatment Planning

**Figure 1:**
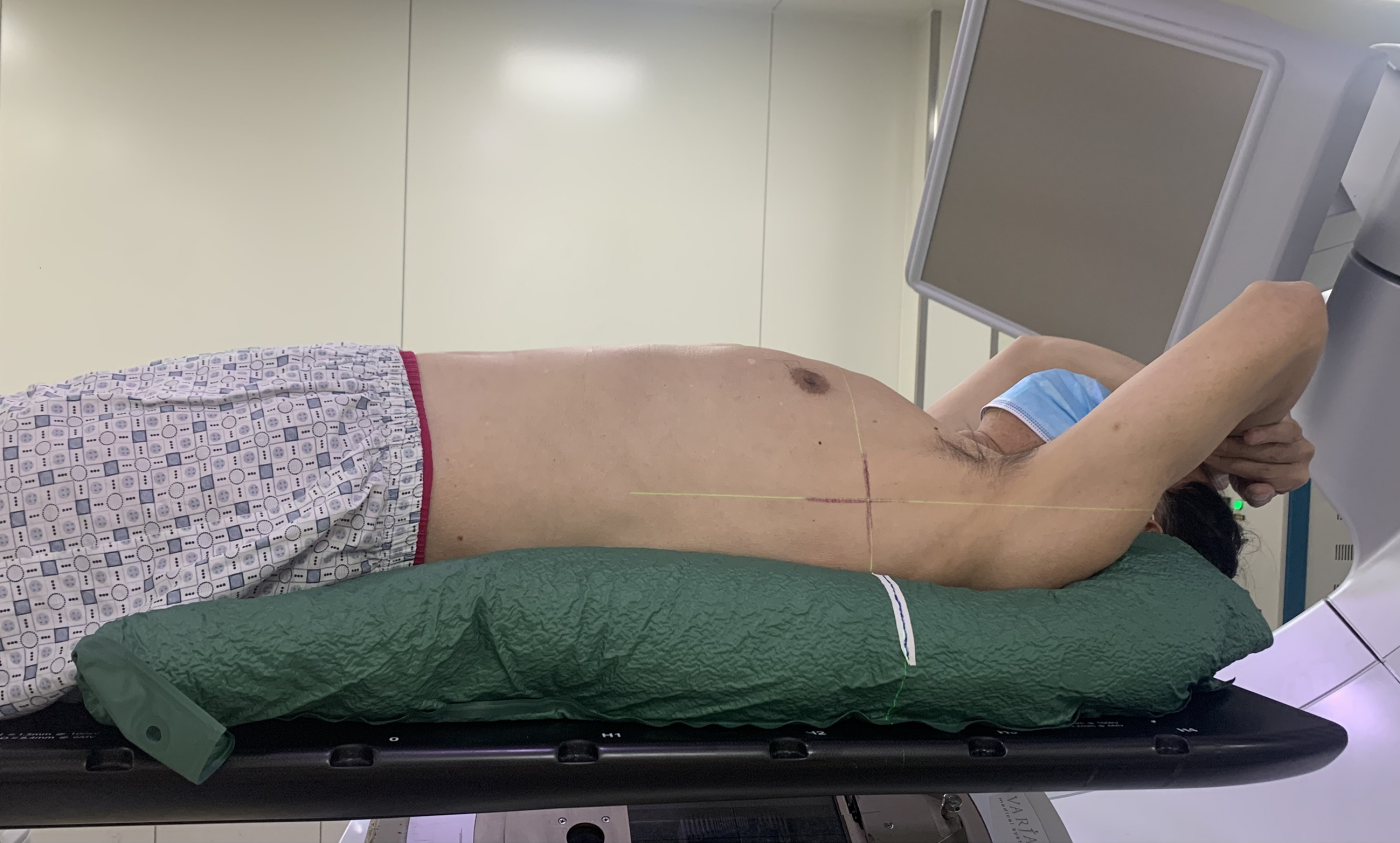
Example of immobilization and localization systems with vacuum cushion. Patient is in the supine position and immobilized by a vacuum cushion with the arm placed above the head during free breathing. The marker on vacuum cushion and the skin marker on the patient are both aligned with the room laser.

Breath training was undertaken before scanning to help patients achieve regular and stable breathing pattern, and the patients were scanned until reproducible patterns of respiration were observed. Each patient had a 4DCT on a CT simulation (Siemens Healthcare, Erlangen, Germany) in supine position and was immobilized using vacuum cushions with arms placed above the head during free breathing (see **Figure 1**). Scanning parameters included 3 mm slice thickness, field of view measuring 45 × 45 cm, 120 kV voltage, and 250 mA X-ray tube current. The 4DCT images were acquired and sorted out into nine respiratory phases based on respiratory cycle signal recorded by the Varian Real-Time Position Management System (VARIAN Medical Systems Inc., USA). The scan encompassed the third cervical spine up to the lower edge of the liver including the whole lung. Nine phases of 4DCT and average CT datasets of each patient were transferred to a commercially deformable image registration tool MIM (MIM Software Inc., Cleveland, OH, USA) for delineation of tumor.

The gross tumor volume (GTV) was contoured manually on nine phases of the 4DCT scan, and nine GTVs were then combined to generate ITV on average CT dataset for further contouring and planning. The planning target volume (PTV) expanded 5–6 mm in every direction based on the ITV range. All contours and average CT datasets were then transferred to the Pinnacle^3^ treatment planning system v9.10 (Philips Healthy, Fitchburg, WI). OARs were contoured on the average CT dataset, which was selected for the treatment plan. Each patient received a 10–15 field SBRT plan using coplanar technique three-dimensional conformal radiotherapy (3D-CRT) with four to eight fractions. Dose constraints were within tolerance limits, based on RTOG 0236 protocol (4). Briefly, 100% prescription dose was normalized to cover 95% of the PTV, 99% coverage of the PTV was encompassed by at least 90% of the prescribed dose, and 100% of isodose line was employed to cover 100% of ITV. The treatment planning was delivered on the Edge™ linear accelerator (Varian Medical Systems, Palo Alto, CA).

### Patient Setup and Setup Measurements

**Figure 2:**
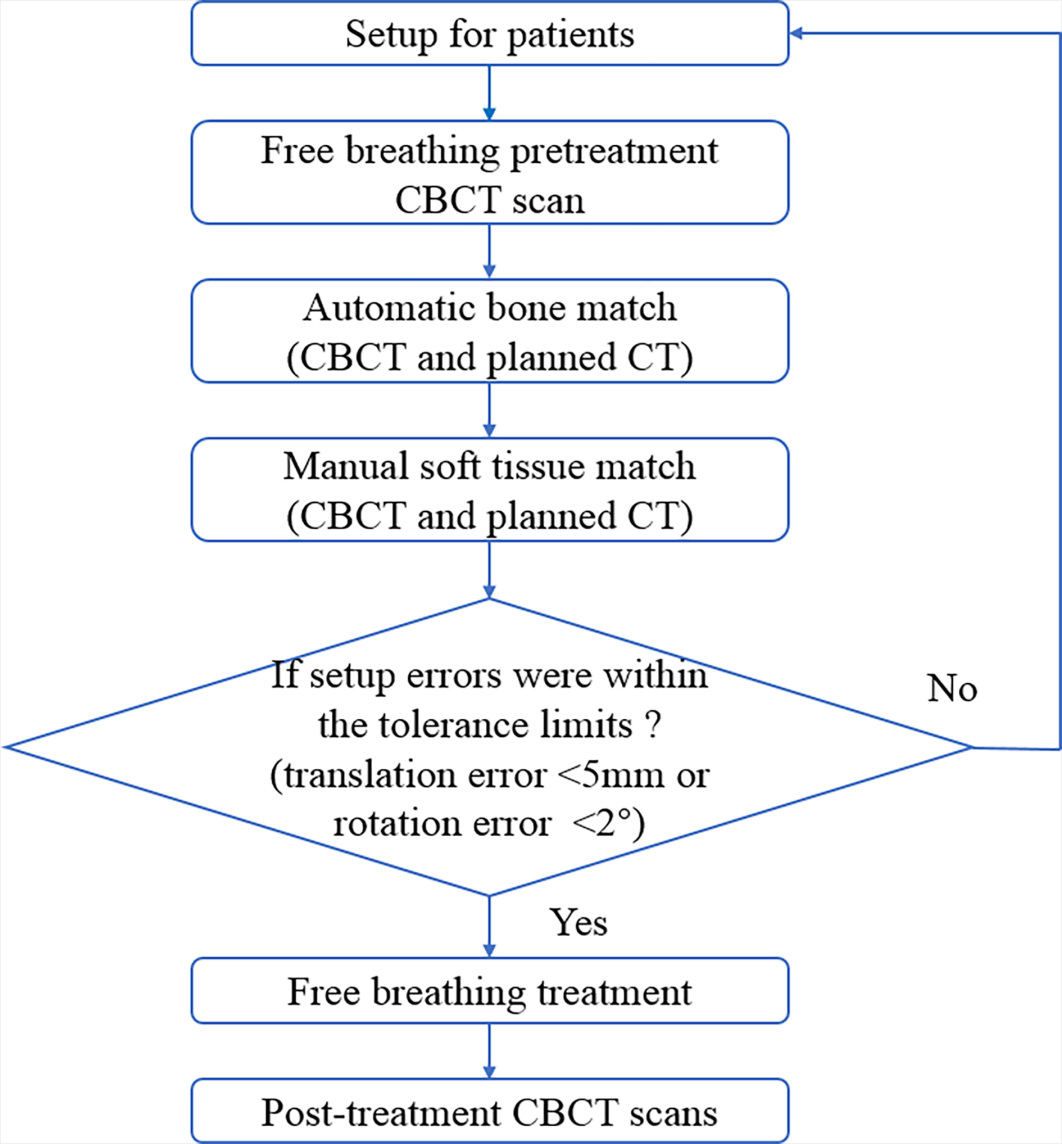
Patients treatment workflow for each treatment fraction. CBCT, cone-beam computed tomography.

The workflow for one fraction treatment is described in **Figure 2**. Before each treatment fraction, patients were initially setup using in-room lasers and reference skin tattoos, and a free breathing pretreatment kv-CBCT with a 360° clockwise standard rotation was acquired for all patients. First, CBCT and the planned CT were rigidly registered with the bony anatomy. Manual registration of tumor and soft tissue of the patient was then undertaken, and shifts of the treatment couch were acquired to correct the differences in translational and rotational axes. Based on tolerance of the institute, patient re-setup and repeated CBCT were needed when translation error exceeded 5 mm in any anterior–posterior (AP), superior–inferior (SI), and left–right (LR) directions or when rotation error exceeded 2° in any pitch, roll, and rtn directions. The free breathing treatment was undertaken until both translation and rotation errors were within tolerance limits. Shifts including translation and rotation data were then transferred to 6D robotic couch (Varian Medical Systems Inc., Baden, Switzerland). A posttreatment CBCT was finally undertaken immediately after the end of treatment.

### Statistical Analyses

All interfraction setup errors were reported as mean ± standard deviation (SD). The Shapiro–Wilk test showed that all parameters were normally distributed. Differences between groups were analyzed using independent sample t-test.

Logistic regression test was used to quantify effects of the clinical and tumor factors over setup errors, in which dependent variable was patient re-setup, and independent variables included all factors presented in **Tables 1** and **2**. Findings are shown in OR with 95% CI.

All data analyses were undertaken using SPSS Statistics v22.0 software (IBM Corp., Armonk, NY, USA). p <0.05 was considered statistically significant.

## Results

### Patient Interfraction Setup Errors

**Figure 3:**
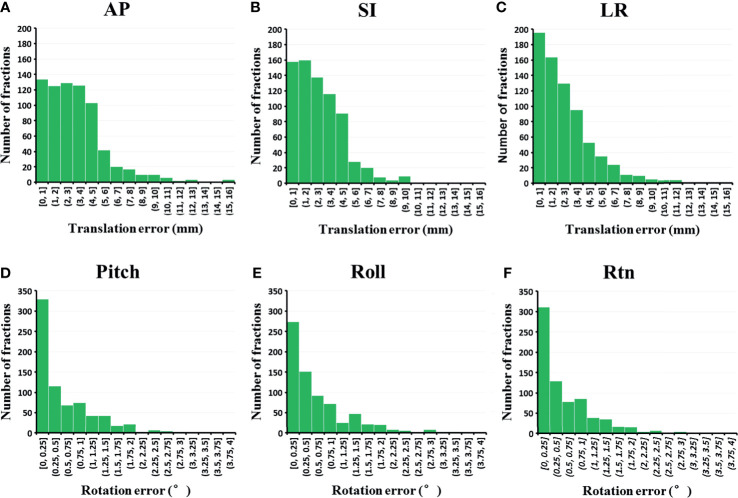
Distribution of the interfraction setup errors in **(A)** AP, **(B)** SI, **(C)** LR, **(D)** pitch, **(E)** roll and **(F)** rtn direction (n = 732 fractions). AP, anterior-posterior; LR, left-right.

A total of 732 predelivery CBCT images were collected for 142 patients. Mean and maximum initial setup errors for patients are shown in **Table 3**. For translation setup errors, the mean setup error was largest in the AP direction (3.2 ± 2.4 mm), and the maximum setup error was also larger in this direction (6.7 ± 3.5 mm) compared with the setup in SI (5.8 ± 2.9 mm) and LR (5.1 ± 2.9 mm) directions. Regarding rotation setup errors, the mean and maximum values were similar in pitch, roll, and rtn directions. **Figure 3** shows distribution of interfraction setup errors in different directions.

**Table 3:** The initial setup errors analysis of 732 times of CBCT scan for 142 cases.

Direction	Mean	Maximum
AP (mm)	3.2 ± 2.4	6.7 ± 3.5
SI (mm)	2.8 ± 2.1	5.8 ± 2.9
LR (mm)	2.5 ± 2.0	5.1 ± 2.9
Pitch (°)	0.5 ± 0.6	1.0 ± 0.7
Roll (°)	0.6 ± 0.7	1.1 ± 0.8
Rtn (°)	0.5 ± 0.6	1.1 ± 0.7

CBCT, cone-beam computed tomography; AP, anterior–posterior; SI, superior–inferior; LR, left–right.

**Figure 4:**
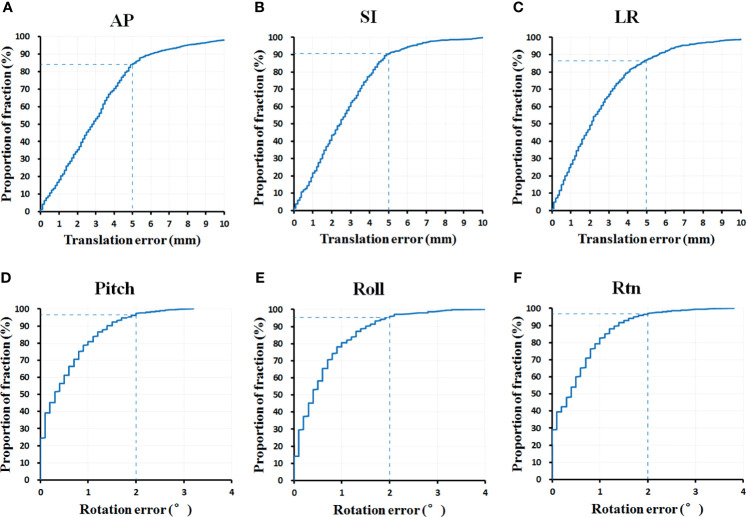
The percentage of fractions out of setup error tolerance limits in **(A)** AP, **(B)** SI, **(C)** LR, **(D)** pitch, **(E)** roll and **(F)** rtn direction (n = 732 fractions). AP, anterior-posterior; SI, superior-inferior; LR, left-right.

The percentage of fractions out of setup error tolerance limits (translation errors >5 mm in any AP, SI, and LR direction or rotation errors >2° in any pith, roll, and rtn directions) for initial CBCT were also computed in the current study. Of the total 732 CBCT fractions, 83.7%, 90.3%, and 86.6% satisfied setup error tolerance limits in AP, SI, and LR directions, respectively (see **Figure 4**). In addition, 95% of the total fractions had rotation setup errors of <2° in pitch, roll, or rtn directions.

### Clinical and Tumor Factors Influencing the Interfraction Setup Error

Findings of independent sample t-test showed that the patient group of age ≤68 had significantly higher setup error in LR direction (p = 0.038) and significantly lower setup error in pitch direction (p = 0.025) compared with the patient group of age >68 with no differences in other directions (see **Table 4**).

**Table 4:** Group comparison for clinical and tumor characteristics.

Direction	AP (mm)	SI (mm)	LR (mm)	Pitch (°)	Roll (°)	rtn (°)	p-value
**Age (years)**							
≤68	3.2 ± 2.3	2.7 ± 2.1	2.2 ± 2.0*	0.6 ± 0.5*	0.6 ± 0.6	0.5 ± 0.6	LR: 0.038
>68	3.3 ± 2.5	2.8 ± 2.0	2.9 ± 2.1	0.4 ± 0.5	0.6 ± 0.6	0.6 ± 0.6	Pitch: 0.025
**Gender**							
Male	3.2 ± 2.2	2.9 ± 2.1	2.4 ± 2.0	0.6 ± 0.6	0.6 ± 0.6	0.5 ± 0.5	
Female	3.3 ± 2.3	2.8 ± 2.4	2.6 ± 1.9	0.5 ± 0.6	0.7 ± 0.8	0.6 ± 0.7	
**BMI**							
BMI ≤ 18.5	3.3 ± 2.3	2.9 ± 2.0	1.8 ± 1.4*	0.4 ± 0.5	0.7 ± 0.8	0.4 ± 0.4*	
18.5 < BMI ≤ 24	3.3 ± 2.4	2.7 ± 2.3	2.4 ± 2.0	0.6 ± 0.6	0.6 ± 0.6	0.5 ± 0.6	LR: 0.018
BMI > 24	3.2 ± 2.3	2.8 ± 2.2	2.7 ± 2.1	0.5 ± 0.6	0.6 ± 0.7	0.6 ± 0.7	Rtn: 0.007
**Ability to speak Mandarin**							
No	3.3 ± 2.3	2.8 ± 2.0	2.5 ± 1.8	0.6 ± 0.5	0.6 ± 0.6	0.6 ± 0.5	
Yes	3.2 ± 2.3	2.9 ± 2.2	2.4 ± 2.0	0.5 ± 0.5	0.6 ± 0.6	0.5 ± 0.6	
**Education level**							
Illiterate	3.1 ± 2.5	2.8 ± 2.4	2.5 ± 2.1	0.6 ± 0.5	0.8 ± 0.7	0.6 ± 0.5	
Primary school	3.2 ± 2.7	2.9 ± 2.1	2.6 ± 2.3	0.5 ± 0.6	0.6 ± 0.6	0.5 ± 0.6	
Middle school	3.3 ± 2.5	2.8 ± 2.3	2.3 ± 2.0	0.6 ± 0.6	0.5 ± 0.6	0.6 ± 0.6	
High school	3.1 ± 2.8	2.7 ± 2.6	2.6 ± 1.9	0.4 ± 0.5	0.6 ± 0.5	0.4 ± 0.4	
University qualifications	2.4 ± 2.3	2.9 ± 2.2	2.5 ± 2.0	0.5 ± 0.5	0.6 ± 0.6	0.5 ± 0.6	
**Location**							
Right	3.3 ± 2.9	2.9 ± 2.3	2.4 ± 2.0*	0.4 ± 0.5*	0.6 ± 0.6	0.5 ± 0.6	LR: 0.027
Left	3.2 ± 2.8	2.7 ± 2.1	2.7 ± 2.2	0.6 ± 0.7	0.7 ± 0.7	0.6 ± 0.6	Pitch: 0.003
**Location**							
Upper	3.2 ± 2.6	2.8 ± 2.5	2.6 ± 2.3	0.5 ± 0.6	0.6 ± 0.7	0.6 ± 0.6	
Middle	3.3 ± 2.2	2.9 ± 2.5	2.3 ± 2.0	0.5 ± 0.5	0.6 ± 0.6	0.5 ± 0.6	
Lower	3.2 ± 2.5	2.9 ± 2.4	2.4 ± 2.0	0.6 ± 0.5	0.7 ± 0.7	0.5 ± 0.6	
**Maximum diameter of ITV (cm)**							
≤3	3.3 ± 2.3	2.8 ± 2.4	2.5 ± 2.0	0.5 ± 0.6	0.6 ± 0.6	0.5 ± 0.6	
>3	3.2 ± 2.1	2.7 ± 2.3	2.4 ± 2.2	0.6 ± 0.6	0.6 ± 0.5	0.6 ± 0.7	
**Volume of ITV (cm^3^)**							
≤7.9	3.2 ± 2.0	2.8 ± 2.2	2.4 ± 2.3	0.6 ± 0.6	0.6 ± 0.6	0.5 ± 0.6	
>7.9	3.2 ± 2.1	2.7 ± 2.2	2.5 ± 2.2	0.5 ± 0.6	0.6 ± 0.7	0.5 ± 0.5	
**Distance from ITV boundary to vertebral body boundary (cm)**							
≤3.8	3.1 ± 2.5	2.8 ± 2.0	2.4 ± 1.7	0.5 ± 0.6	0.6 ± 0.7	0.5 ± 0.6	
>3.8	3.3 ± 2.7	2.8 ± 2.1	2.6 ± 2.0	0.6 ± 0.6	0.6 ± 0.6	0.6 ± 0.6	
**Distance from ITV boundary to heart boundary (cm)**							
≤3.8	3.2 ± 2.7	2.9 ± 2.2	2.6 ± 2.4	0.5 ± 0.6	0.7 ± 0.7	0.6 ± 0.7	
>3.8	3.3 ± 2.5	2.8 ± 2.4	2.5 ± 2.1	0.6 ± 0.6	0.6 ± 0.6	0.5 ± 0.5	
**Distance from ITV boundary to chest wall boundary (cm)**							
≤1	3.2 ± 2.6	2.9 ± 2.4	2.5 ± 2.4	0.6 ± 0.6	0.6 ± 0.7	0.6 ± 0.6	
>1	3.4 ± 2.7	2.7 ± 2.1	2.3 ± 2.0	0.5 ± 0.6	0.7 ± 0.7	0.5 ± 0.6	

*Indicates a statistically significant difference.

BMI, body mass index; AP, anterior–posterior; SI, superior–inferior; LR, left–right; ITV, internal target volume.

BMI findings showed a significant relationship with setup errors in the LR direction, showing 1.8 ± 1.4 mm, 2.4 ± 2.0 mm, and 2.7 ± 2.1 mm for patients with BMI ≤ 18.5, 18.5 < BMI ≤ 24, and BMI > 24, respectively (p = 0.018). Setup errors in rtn direction between the three BMI groups were also significantly different (p = 0.007) (see **Table 4**).

The mean setup errors in LR and pitch directions were both significantly higher in left side lesions (p = 0.027, p = 0.003, respectively) compared with the right side lesions.

Notably, all group comparisons of the three factors showed statistically significant differences in the LR direction.

Other factors including “upper *vs.* middle *vs.* lower” location, maximum diameter of ITV, volume of ITV, gender, ability to speak Mandarin, education level, distance from ITV boundary to vertebral body boundary, distance from ITV boundary to heart boundary, and distance from ITV boundary to chest wall boundary had no significant correlations with the setup error.

### Correlation Between Interfraction Setup Errors and Clinical and Tumor Characteristics

Bivariate logistic regression models were used to explore possible correlations between clinical and tumor factors with patient re-setup in lung SBRT immobilized using VC. Single variation analysis showed that only age (p = 0.003) and BMI (p < 0.001) correlated with patient re-setup (see **Table 5**). Multivariable model analysis showed that age (p = 0.006) and BMI (p = 0.002) also affected patient re-setup, and other factors had no impact on patient re-setup.

**Table 5:** Correlation between patient re-setup with the clinical and tumor factors (translation error >5mm or rotation error >2° in any direction).

Factor	Single predictor			Multi-predictor		
	OR	95%CI	p	OR	95%CI	p
**Age**	2.87	1.45–5.68	0.003*	3.25	1.39–7.58	0.006*
**Gender**					
Male	1			1		
Female	1.06	0.54–2.08	0.862	0.73	0.30–1.76	0.481
**BMI**	3.89	1.93–7.83	<0.001*	3.77	1.70–8.35	0.002*
**Can understand Mandarin**						
No	1			1		
Yes	1.91	0.84–4.33	0.122	2.35	0.82–6.69	0.111
**Education level**					
Illiterate	1			1		
Primary school	2.10	0.69–6.35	0.189	0.89	0.24–3.32	0.873
Middle school	2.33	0.79–6.98	0.130	0.62	0.15–2.59	0.512
High school	1.14	0.37–3.47	0.821	0.26	0.06–1.16	0.078
University qualifications	1.02	0.29–3.65	0.975	0.34	0.06–1.86	0.213
**Location**					
Left	1			1		
Right	1.90	0.97–3.71	0.060	1.77	0.80–3.94	0.160
**Location**					
Upper	1			1		
Middle	3.50	1.21–5.12	0.562	2.33	0.62–8.88	0.213
Lower	1.32	0.63–2.76	0.460	1.25	0.48–3.24	0.654
**Maximum diameter of ITV(cm)**	0.57	0.29–1.10	0.094	0.59	0.03–13.86	0.743
**Volume of ITV (cm^3^)**	0.58	0.19–1.01	0.087	1.10	0.05–25.95	0.953
**Distance from ITV boundary to vertebral body boundary**	1.41	0.73–2.73	0.313	2.01	0.89–4.51	0.093
**Distance from ITV boundary to heart boundary**	1.12	0.58–2.17	0.737	0.87	0.35–2.14	0.757
**Distance from ITV boundary to chest wall boundary**	1.25	0.70–2.55	0.845	0.99	0.43–2.33	0.995

*Indicates a statistically significant difference.

BMI, body mass index; ITV, internal target volume.

## Discussion

The current study evaluated interfraction translation and rotation setup errors in lung SBRT patients immobilized in the vacuum cushions device and analyzed clinical and tumor factors that influence patient re-setup. Findings showed that age and BMI had significant correlation with patient re-setup, whereas right *versus* left location showed a trend to affect setup error.

In lung SBRT, adjacent critical structures are close to the target and the fractional high doses and steep dose gradient; precise and reproducible interfractional setup is critical. Currently, several immobilization systems are used, some of which are customized, although there is no clear standard immobilization method so far. Studies have indicated that different immobilization systems generate different inter- and intrafraction setup errors (15–17). However, findings of other studies have different conclusions. Siva et al. established that VC system allowed for highly reproducible patient positioning and robust intrafraction patient immobilization (21). Nielsen et al. showed that setup uncertainties at the two institutions were the same despite different fixation equipment (22). Therefore, evaluation of the immobilization device for an institution is needed. In our institution, the immobilization devices used for lung SBRT are almost exclusively VCs, with occasional special cases of patients using thermoplastic masks. In addition, patient re-setups are undertaken before treatment with a translation error of >5 mm in any one direction or a rotation error of >2° in any one direction until the setup error meets the limit requirement. Clinically, the current study indicated that the number of patient re-setups is still relatively high, which increases treatment time of patients and workload of technologists. This is a disadvantage for a busy institution, and re-setup increases treatment cost. Therefore, analysis of factors influencing re-setup is necessary to provide constructive guidance suggestions for clinical setup. In the current study, interfraction setup errors in the 6D direction and the number of patient re-setup for patient with VC device were computed, and the clinical and tumor factors that may influence patient re-setup were analyzed. The current study envisages that the findings will provide guidance for clinical patient setup and offer reference for other institutes that use or plan to use VCs.

CBCT 6D/6D match offered the most accurate patient positioning in both translations and rotations. Combined with the 6D couch, translational and rotational setup errors can be minimized effectively (23). In the current study, translational and rotational setup errors for lung SBRT were both corrected. Interfraction translation setup errors in AP, SI, and LR directions were 3.2 ± 2.4, 2.8 ± 2.1, and 2.5 ± 2.0 mm, respectively, whereas rotation errors in pitch, roll, and rtn directions were 0.5 ± 0.6°, 0.6 ± 0.7°, and 0.5 ± 0.6°, respectively. Chen et al. established that the interfraction setup errors with VC were 2.4 ± 2.1, 2.2 ± 2.1, and 2.2 ± 2.0 mm in AP, SI, and LR directions, respectively (15), which were less compared with findings of the present study. This may be explained by differences in institutional regulations. Chen et al. first corrected the translation setup error using a rigid registration of the body anatomy and then corrected the translation setup errors using manual registration of the ITV contour. In the current study, both translation and rotation setup errors were corrected simultaneously.

In the current study, 16.3% of fractions had setup errors more than 5mm in AP direction, whereas 9.7% and 13.4% of fractions had setup errors in SI and LR directions. These findings indicate that the number of patient re-setups for translation error was still relatively high, but it was better for rotation error. Furthermore, these findings differed with those of Chen et al., indicating that the current study was more stringent about rotational setup error. Different institutions may have different requirements for patient re-setup (20, 24), but the goal is to ensure as precise and reproducible setup as possible for SBRT patients.

To explore possible factors influencing the setup error, both clinical and tumor factors were analyzed in the current study. Findings of group analysis showed that setup errors in LR and pitch directions were higher in older patients. The group with larger BMI had higher setup errors in the LR and rtn directions compared with the group with smaller BMI. Moreover, findings of univariate and multivariate analyses showed that both age and BMI had significant influences on patient re-setup. Liu et al. and Shah et al. observed that age or BMI had no impact on large intrafraction shift (25, 26). A similar conclusion was reached by Chen et al. on the interfraction setup error (15). However, Rico et al. averred that age was the only factor that significantly influenced intrafraction variation (17). The reason for correlation between age and interfraction setup error is unknown, although it may be explained by greater involuntary movements with older patients when using VC device. Self-control of older patients is relatively weak and is difficult to maintain a position all the time. The space available for the patient to move is larger for VC device, and it is possible that the patient slightly adjusted the position when the technician left. The effect of BMI on interfraction setup error has been demonstrated in several previous studies (15, 27). These findings are consistent with findings of the current study. In the present study, the setup error differed only in the “‘right *vs.* left” position but not in the “upper *vs.* middle *vs.* lower” position. In addition, findings of logical analysis showed that “right *vs.* left” position had no influence on patient re-setup. The current study considered that left lesions are probably close to the heart, and heartbeat may disturb image matching.

However, the current retrospective study had some limitations. First, the present study evaluated only interfraction setup error, but not intrafraction shift, which was occasioned by resource limitation. Second, the current study did not consider positional technique differences among technicians. Finally, other clinical factors, including imaging protocol and abdominal compression that may have influences on setup errors, were not evaluated. The current study recommends inclusion of these factors in future studies.

## Conclusion

The current study established that age and BMI of clinical factors were correlated with patient re-setup in NSCLC SBRT, whereas all other clinical and tumor factors were not correlated with patient re-setup. Based on findings of the current study, it is recommended that immobilization of older patients and patients with larger BMI should be considered.

## Data Availability Statement

The raw data supporting the conclusions of this article will be made available by the authors, without undue reservation.

## Ethics Statement

The studies involving human participants were reviewed and approved by the ethics committee of Shanghai Chest Hospital. The patients/participants provided their written informed consent to participate in this study.

## Author Contributions

HC wrote the manuscript. HC and YH analyzed the data. All authors participated in the design of the presented study and reviewed the manuscript prior to its publication.

## Funding

This work was sponsored by the Interdisciplinary Program of Shanghai Jiao Tong University (Grant No. YG2019ZDB07) and Nurture Projects for Basic Research of Shanghai Chest Hospital (Grant No. 2019YNJCM05).

## Conflict of Interest

The authors declare that the research was conducted in the absence of any commercial or financial relationships that could be construed as a potential conflict of interest.

## Publisher’s Note

All claims expressed in this article are solely those of the authors and do not necessarily represent those of their affiliated organizations, or those of the publisher, the editors and the reviewers. Any product that may be evaluated in this article, or claim that may be made by its manufacturer, is not guaranteed or endorsed by the publisher.
